# Tumor hypoxia unveiled: insights into microenvironment, detection tools and emerging therapies

**DOI:** 10.1007/s10238-024-01501-1

**Published:** 2024-10-03

**Authors:** Joanna Ciepła, Ryszard Smolarczyk

**Affiliations:** Center for Translational Research and Molecular Biology of Cancer, Maria Skłodowska-Curie National Research Institute of Oncology, Gliwice Branch, Wybrzeże Armii Krajowej Street 15, 44-102 Gliwice, Poland

**Keywords:** Cancer, Hypoxia, Tumor microenvironment, Anti-cancer therapy, Detection

## Abstract

Hypoxia is one of the defining characteristics of the tumor microenvironment (TME) in solid cancers. It has a major impact on the growth and spread of malignant cells as well as their resistance to common treatments like radiation and chemotherapy. Here, we explore the complex functions of hypoxia in the TME and investigate its effects on angiogenesis, immunological evasion, and cancer cell metabolism. For prognostic and therapeutic reasons, hypoxia identification is critical, and recent developments in imaging and molecular methods have enhanced our capacity to precisely locate underoxygenated areas inside tumors. Furthermore, targeted therapies that take advantage of hypoxia provide a potential new direction in the treatment of cancer. Therapeutic approaches that specifically target hypoxic conditions in tumors without causing adverse effects are being led by hypoxia-targeted nanocarriers and hypoxia-activated prodrugs (HAPs). This review provides an extensive overview of this dynamic and clinically significant area of oncology research by synthesizing current knowledge about the mechanisms of hypoxia in cancer, highlighting state-of-the-art detection methodologies, and assessing the potential and efficacy of hypoxia-targeted therapies.

## Introduction

Solid tumors are one of the most common forms of cancer, characterized by high morbidity and mortality levels among patients [134]. They are a heterogeneous mass of infiltrating and resident host cells, secreted factors, and extracellular matrix (ECM). Unlike normal tissues, the demand for oxygen in solid tumors becomes inadequate due to the uncontrollably growing mass. To compensate, tumors develop their own vascular system through the process of angiogenesis. This usually appears when the tumor exceeds a diameter of approximately 1 mm [42]. Although functional, the vessels are chaotic, primitive, and display various structural abnormalities [128]. Such leaky, twisted and blind-ended blood vasculature is unable to meet the metabolic needs for development, leading to the formation of areas displaying low oxygen, glucose and energy, along with high lactate, extracellular acidity levels, and oxygen deficiency. These microenvironmental changes trigger a cascade of cellular and molecular responses promoting tumor aggressiveness, angiogenesis, and immune evasion [71, 88]. One of the factors that stimulate such a response is hypoxia.

## Tumor hypoxia

**Fig. 1:**
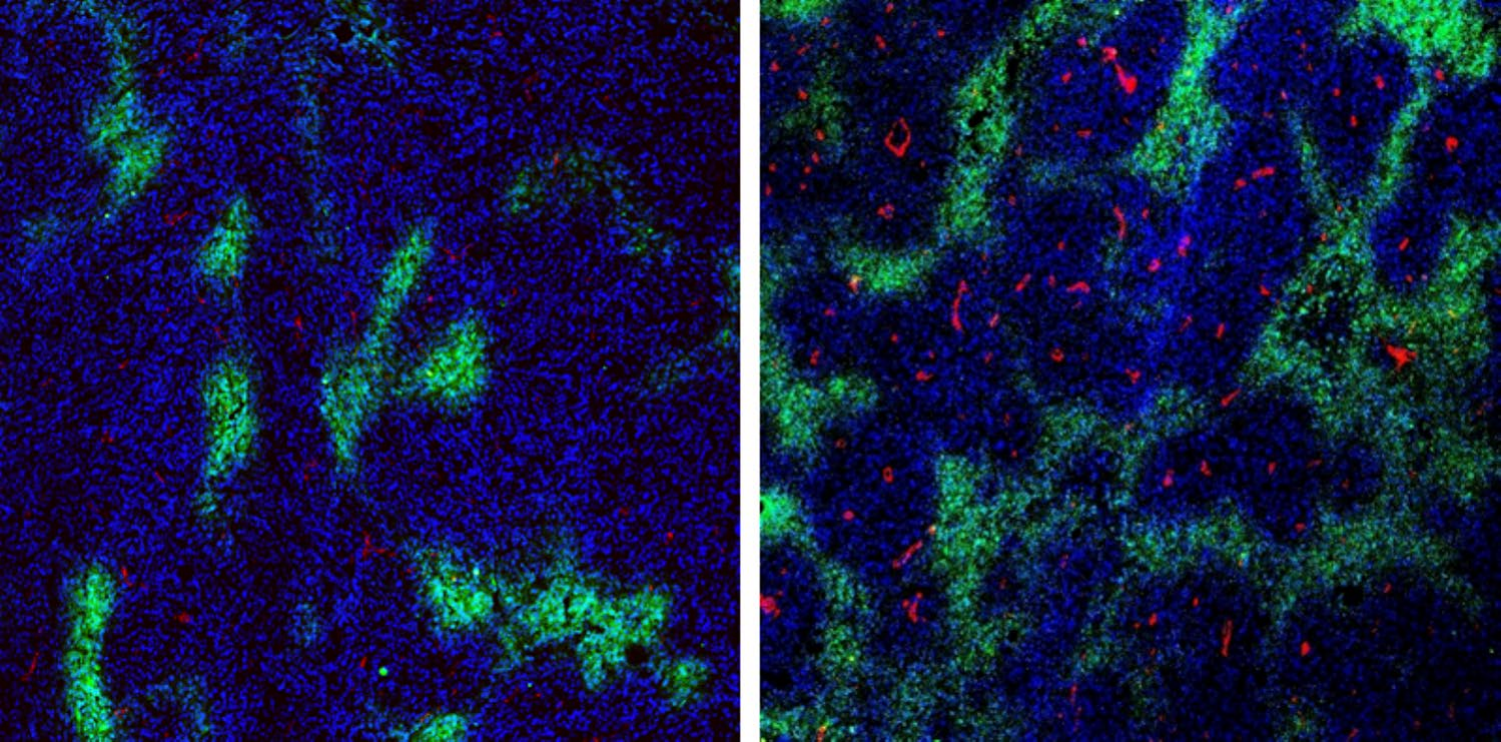
Immunofluorescence staining image of endothelial cells and hypoxia, CD31 positive—endothelial cells (red), pimonidazole—hypoxia (green), nucleus (blue) in murine breast cancer—4T1 tumor tissues. Tumors of different sizes. Smaller on the left, larger on the right

Hypoxia (regions of underoxygenation) is one of the most frequent hallmarks of solid tumors, appearing in 90% of cases. As the tumor grows, the number of hypoxia regions increases and occupies a significant area of the tumor (Fig. 1). Normal tissues exist at 2–9% O_2_ (on average 40 mm Hg). Hypoxic microenvironment varies between 0.02 and 2% O_2_ (below 10 mm Hg); while, severe hypoxia or “anoxia” is defined as ≤ 0.02% O_2_ [10]. Hypoxia is closely associated with malignant progression, increased distant tumor metastases, heightened aggressiveness, resistance to various types of therapies, and an overall poor prognosis [113]. Regions of the primary tumors may be exposed to various types of hypoxia, such as acute, chronic or intermittent. Acute hypoxia is described as short-term (no longer than 24 h) exposure to oxygen levels below 1% that can be reversed with returned blood flow; while, chronic hypoxia is characterized by long exposure to an environment of less than 1% O_2_. Hypoxia can also occur in a cycling or intermittent phase which arises due to the temporary shutdown of flawed tumor vasculature resulting in transient blood flow. Intermittent hypoxia (IH) is caused by exposure to cycles of hypoxia and reoxygenation (H–R cycles) [6, 33, 37].

As the tumor microenvironment (TME) becomes more hypoxic, cells employ certain stress responses as a mechanism of survival [76]. Oncogenes and tumor suppressors mediate these stress responses by activating specific molecules and signaling pathways crucial to the regulation of aerobic glycolysis [123]. Cancer cells adapt to hypoxic conditions by inducing both proteomic and genomic changes. Proteomic changes may initiate cell cycle arrest or terminal differentiation, leading to necrotic death; however, they may also stimulate tumor growth and metastases by promoting acclimatization and survival in a hostile, nutrient-deprived environment. The development of tumor aggressiveness is strongly influenced by mutations in oncogenes and/or tumor suppressor genes [144]. Hypoxia promotes genomic instability due to increased production of reactive oxygen species (ROS) and alterations in the DNA damage repair pathways, thereby increasing the occurrence of these mutations [62]. The expansion of cell clones with favorable proteomic and genomic adaptive changes can, in turn, exacerbate tumor hypoxia, thereby establishing a vicious cycle of increasing hypoxia and subsequent malignant progression [144]. Other hypoxia-induced responses include: upregulation of vascular endothelial growth factor (VEGF) expression, which stimulates the growth of new tumor blood vessels, promotion of autophagy, and stimulation of the epithelial–mesenchymal transition (EMT). In addition, hypoxia creates disadvantageous microenvironments for immune cells and dampens immune response-related signals, inducing immunosuppression [21].

Despite significant research, major gaps remain in our understanding of hypoxia’s role in tumor growth, metastasis, and the immune suppression necessary for tumor survival. Increasing evidence suggests that normalizing tumor blood vessels can enhance therapeutic efficacy, improving oxygenation, drug delivery, and reducing tumor resistance to radiotherapy and chemotherapy. However, the role of vessel normalization in immunotherapy, a widely used treatment in clinical and experimental settings, is less understood. Functional blood vessels in tumors are expected to facilitate immune cell infiltration, which is crucial for tumor destruction.

It is crucial to understand the mechanisms underlying hypoxia in cancer and how it affects tumor biology in order to design efficient therapeutic strategies. The aim of this review is to investigate the various aspects of hypoxia in cancer, with a particular emphasis on its affects the TME, detection methods and its potential as a therapeutic target.

## Hypoxia-induced transcription factors

Transcription factors induced by hypoxia play a pivotal role in orchestrating cellular responses to low-oxygen conditions within the TME. Hypoxia, a hallmark feature of solid tumors, triggers a complex molecular adaptive response largely governed by the activation of key transcription factors. These transcription factors, such as hypoxia-inducible factor (HIF), factor inhibiting HIF-1 (FIH-1), and nuclear factor-κB (NF-κB), not only regulate the expression of genes involved in cell survival and metabolism but also modulate various aspects of tumor biology, such as angiogenesis, immune evasion, and therapeutic resistance [7, 137].

### HIF

The most significant and best understood mechanism of tumor cells’ adaptation to hypoxic stress is regulation by HIF, a transcription factor which accumulates in response to decreased cellular oxygen level.

The concept that the effects of hypoxia on tumor cells are primarily mediated by the HIF family was first described in 1990s [127, 152]. These transcription factors play a fundamental role in metabolic adaptation to hypoxia, and promote the expression of more than 150 genes, including VEGF, erythropoietin, transferrin and transferrin receptors, enzymes crucial for glycolysis, anti-apoptotic factors, various growth factors, and other proteins that preserve homeostasis and promote proliferation, invasion, and metastasis [100].

HIF consists of a cytoplasmic O_2_-sensitive *α* subunit (HIF-1*α*, HIF-2*α* and HIF-3*α*) that has a half-life of minutes, and *β* subunit that is constitutively expressed and independent of oxygen levels. HIF-1*α* and HIF-2*α* play essential roles in positive hypoxic response; while, HIF-3*α* is suggested to be a negative modulator [156]. The accumulation of individual HIF*α* subunits depends on the duration of hypoxia. HIF-1*α* is activated in the first 4 h of hypoxia, after which protein levels drastically decrease. As opposed to HIF-2*α* and HIF-3*α* reaching their maximum levels after 24–48 h in hypoxic environment [116].

In normoxia, when oxygen supply is sufficient, proline hydroxylase domain protein 1–3 (PHD1-3) hydroxylates oxygen-dependent degradation domain (ODD) of the HIF-1*α* subunit [44, 60]. The hydroxylated prolyl sites are recognized by the Von Hippel–Lindau (VHL) tumor suppressor, which induces degradation of HIF-1*α* by the ubiquitin–proteasome system (UPS) [104]. However, under hypoxic conditions, the rate of hydroxylation and ubiquitination decreases, resulting in accumulation of non-hydroxylated HIF-1*α* subunit, which then translocates to the nucleus, where it combines with the HIF-1*β* subunit forming heterodimer. This composed heterodimer is an active HIF-1 factor which binds to the hypoxia response elements (HRE) of target genes driving transcriptional responses of hundreds of genes including those for cell proliferation, metastasis, glycolysis, pH regulation and angiogenesis [69, 93, 125].

HIF-1*α* and HIF-2*α* are the main mediators of acute and chronic hypoxia, respectively, and have been reportedly associated with increased tumor aggressiveness and progression [63]. Furthermore, studies have shown acute hypoxia to elevate cell survival and autophagy, selecting stem-like cancer cells and increasing radioresistance [11, 64, 120].

HIF-2*α*, in contrast to HIF-3*α*, displays a significant amount of amino acid sequence similarity to HIF-1*α*, except for the transactivation domain. This identity resemblance may account for their shared ability to heterodimerize with HIF-1*β* and bind to the HREs, which makes them both overlapping and unique target genes. It is interesting to note that the tissue distribution patterns of HIF-1*α* and HIF-2*α* differ. While HIF-1*α* is present throughout the body, HIF-2*α* is only expressed in a limited number of tissues [92]. Furthermore, while HIF-2*α* is primarily engaged in promoting an undifferentiated phenotype in pluripotent cells and promotes chronic hypoxia response (more than 24 h), HIF-1*α* is a key regulator of the glycolytic pathway and is mostly produced during the acute phase of the hypoxia response (less than 24 h) [115].

Reoxygenated tumor cells, after exposure to hypoxia, restart replication combined with impaired repair capacity leading to extensive DNA damage and genomic instability [109]. A study on a murine model of breast cancer demonstrated that acute hypoxia increases metastatic seeding and outgrowth in lungs in vivo. Additionally, subjecting breast tumor cells to cycling hypoxia increased the expression of stem-like cell markers, metastasis-associated gene expression, clonal diversity, and the frequency of tumor-initiating cells. This study shows that, in contrast to chronic hypoxia, acute hypoxia causes a variety of genetic, molecular, biochemical, and cellular changes promoting tumor cell survival, colonization, and the development of a favorable microenvironment, which in turn promotes metastasis [17].

### Factor inhibiting HIF-1 (FIH)

In addition to O_2_, FIH-1 adds another layer of control by hydroxylating asparaginyl residues in HIF-1*α* and HIF-2*α*, preventing the HIF*α* transactivation domain (CAD) from interactions with coactivators like P300 to create a functional transcriptional complex [126]. This pathway represents modifications of HIF-1*α* transactivation domain which does not involve the von Hippel–Lindau (pVHL) protein.

FIH-1 displays a higher affinity for oxygen than PHDs, making it more active under hypoxic conditions. Both FIH-1 and PHDs, in addition to oxygen, require *α*-ketoglutarate (*α*-KG) as a limiting electron donor, Fe^2+^ and ascorbate serving as cofactors for hydroxylation reactions [136]. Limited O_2_ availability results in a significant PHD- and FIH- dependent decrease in HIF-1*α* hydroxylation, leading to HIF-1*α* stabilization and nuclear translocation [136].

### Nuclear factor-κB (NF-кB)

Since numerous transcription factors have been demonstrated to react upon exposure to the hypoxic microenvironment, the cellular response to hypoxia does not solely depend on HIF. NF-κB family of transcription factors composed of RelA, RelB, cRel, NF-κB1 (p105/p50) and NF-κB2 (p100/p52) is one of them. The most conservative pathway of NF-κB activates the Transforming Growth Factor-B activating Kinase-B (TAK1) and the Inhibitor of κB Kinase Complex (IKK). However, there are unusual routes that activate NF-κB without the IKK complex and instead act directly on the Inhibitor of κB (IκBs) [50, 107]. Most NF-κB activation pathways require a ligand to attach to a receptor, such as a cytokine or the detection of foreign DNA or RNA [98]. However, hypoxia or decreased oxygen availability are factors that also stimulate the NF-κB pathway. Several mechanisms have been proposed in the process of hypoxia-induced NF-κB activation. Hypoxia-stimulated NF-κB activation has been shown to require Calcium, TAK1 and IKK as shown in details in another review article [31]. Additionally, the involvement of PHDs and FIH proteins in hypoxia-related activation of the NF-κB pathway has been demonstrated [31, 114].

## Hypoxia in TME

The TME is a dynamic and constantly evolving entity consisting of cancer cells, stromal cells, immune cells, endothelial cells (ECs) and ECM. The composition may vary between tumor types however main components, such as immune and stromal cells, blood vessels, and ECM, remain unchanged [4, 34].

Hypoxia severely impacts the TME, leading to altered cellular behaviors and interactions. In solid tumors, acidosis is the main cause of invasion. Malignant cells require glycolysis, even in the presence of oxygen, to survive in non-hypoxic and intermittent hypoxic environments, known as the “Warburg Effect”. While in hypoxic conditions, cancer cells undergo metabolic reprogramming, favoring anaerobic glycolysis over oxidative phosphorylation; a process known as the “Pasteur Effect” [14, 141]. Nevertheless, lactic acid is the final product of glycolysis in both situations, causing a build up inside the cell. Lactic acid must be expelled from the cell by monocarboxylate transporter 4 (MCT-4) in order for the cell to survive [14]. This metabolic switch not only provides energy for tumor growth, but also contributes to the accumulation of lactate outside the tumor cell via activated monocarboxylic acid transports, leading to acidification of the ECM. Low pH is believed to enhance the invasiveness of tumor cells and inhibit cytotoxicity and proliferation of immune effector cells [45, 74]. As a result, the extracellular environment becomes more acidic, which in turn promotes production of ROS and activates NF-κB [45, 74, 114].

**Fig. 2:**
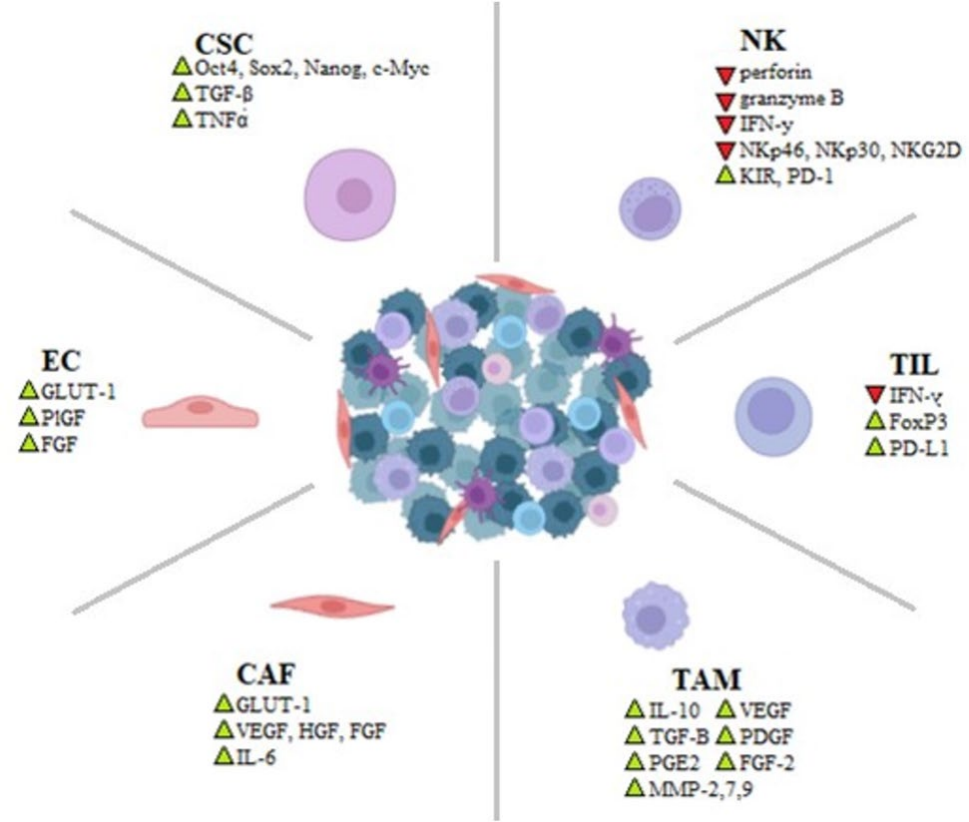
Changes in cytokine secretion by innate immune cells under hypoxic conditions in TME. Hypoxia in tumors regulates the different types of cells present in the tumor microenvironment among others: cancer stem cells (CSC), NK cells (NK), tumor-infiltrating lymphocytes (TIL), tumor-associated macrophages (TAM), cancer-associated fibroblasts (CAF), and endothelial cells (EC)

Furthermore, hypoxia stimulates the recruitment and activation of stromal cells originating from surrounding healthy tissues and from the circulation, such as fibroblasts, macrophages, immune and ECs, which in turn promote tumor angiogenesis, matrix remodeling, and immune evasion [57]. In the stroma of all solid tumors, the major cell populations are cancer-associated fibroblasts (CAFs) and tumor-associated macrophages (TAMs) that frequently perform pro-tumorigenic functions. CAFs and TAMs may work in synergy to promote progression and therapy resistance. The number of CAF or TAM cells found in TME is an indicator of poor prognosis [55] (Fig. 2).

### Cancer-associated fibroblasts (CAFs)

Traditionally, fibroblasts are characterized as non-epithelial, non-immune cells derived from mesenchymal origins, possessing a distinctive elongated shape resembling a spindle. They typically reside in the interstitial spaces or in close proximity to blood vessels. Fibroblasts essential functions are the ability to produce and modify the ECM, regulation of immune cell recruitment, wound healing responses, inflammation, and fibrosis [111].

CAFs are the prominent mesenchymal cell type in the TME playing a major role in the progression of different cancer types. CAFs are formed by the activation or transformation of tumor tissue-resident precursor cells [121]. They alter the surrounding stromal matrix, transforming it into a dense and fibrous structure. Additionally, they release factors that contribute to the development of cancer cells with stem-like properties, support their survival, decrease sensitivity to chemotherapy, and facilitate aggressive tumor growth and metastasis [30]. CAFs contribute to various aspects of TME, including promoting cell proliferation, inflammation, immunosuppression, angiogenesis, invasiveness, and metastasis, which are necessary for the development and advancement of cancer. Based on their phenotypic characteristics, CAFs are categorized into two main groups. The first group consists of CAFs with a myofibroblastic phenotype, referred to as myCAFs. MyCAFs exhibit elevated expression of *α*-smooth muscle actin (*α*-SMA) and fibroblast activation protein (FAP). The second group is known as inflammatory CAFs (iCAFs), which display secretory properties and play a role in regulating inflammation [105].

Hypoxia and ROS contribute to the activation of CAFs). ROS, in particular, have been observed to facilitate the transformation of fibroblasts into myCAFs as a result of the accumulation of HIF-1*α*. Conversely, the use of antioxidants has been found to decrease the levels of HIF-1*α*, thereby inhibiting several properties associated with myCAFs [139]. HIFs, especially HIF-1*α*, play a crucial role in promoting the activation of CAFs. HIF-1*α* upregulates the expression of various cytokines, growth factors, and ECM components, including transforming growth factor-*β* (TGF-*β*), basic fibroblast growth factor (bFGF), platelet-derived growth factor (PDGF), and fibronectin (FN). In a model where CAF formation is induced through TGF-*β* and PDGF treatment, primary CAFs demonstrated an elevated rate of aerobic glycolysis compared to normal fibroblasts. This increase in aerobic glycolysis was found to be linked to the stabilization of the HIF-1*α* protein, suggesting a correlation between CAF activation and the metabolic shift toward aerobic glycolysis [108]. CAFs also secrete pro-angiogenic factors such as VEGF, hepatocyte growth factors (HGF), and fibroblast growth factors (FGF), which contribute to tumor neovascularization [56]. Furthermore, CAFs can suppress the immune response by producing immunosuppressive factors like interleukin-6 (IL-6) affecting dendritic cells maturation, disabling T cell activation. The interplay between CAFs and immune cells in the hypoxic TME creates an immunosuppressive niche that hinders effective anti-tumor immune responses [18, 158]. Hypoxic CAFs exhibit increased glycolytic activity, similar to hypoxic cancer cells, to meet their energy demands. The metabolic reprogramming of CAFs promotes the production of lactate, which not only serves as a metabolic substrate for adjacent cancer cells but also contributes to the acidification of the TME, creating a favorable milieu for tumor invasion and immune evasion. The excess lactate produced by glycolytic tumor cells is removed from the TME through uptake by CAFs, acting as oxidative cells and used as fuel by incorporating it into oxidative phosphorylation in the mitochondria. In contrast to the Warburg effect, hypoxic CAFs (glycolytic cells) in the microenvironment can export lactate into tumor cells (oxidative cells), which then use the lactate to undergo oxidative phosphorylation. This phenomenon is called the reverse Warburg effect [84, 147].

### Tumor-associated macrophages (TAMs)

TAMs are the most abundant immune cells within tumors and can be polarized into different phenotypes, broadly categorized into two subsets: M1 (pro-inflammatory and anti-tumor) and M2 (anti-inflammatory and pro-tumor) [53, 117]. M1 TAMs are activated by various factors including interferon gamma (IFN-*γ*), lipopolysaccharide, interleukin-1 beta (IL-1*β*), tumor necrosis factor (TNF) and/or granulocyte–macrophage colony-stimulating factor (GM-CSF). They possess the ability to recognize and eliminate cancer cells through phagocytosis and cytotoxicity. Furthermore, they produce pro-inflammatory cytokines that stimulate anti-tumor immune responses. On the other hand, M2 TAMs are induced by T helper 2 (Th2) cytokines like interleukin-4 (IL-4), interleukin-10 (IL-10), interleukin-13 (IL-13), and/or macrophage colony-stimulating factor (M-CSF). These M2 TAMs tend to support tumor growth and contribute to TME remodeling by secretion of growth factors, immunosuppressive factors, pro-angiogenic molecules, and proteases [91]. TAMs display dynamic and heterogeneous natures both within individual tumors and across different neoplasms. This innate heterogeneity allows TAMs to adapt to changes within the TME, and thereby actively coordinate and regulate multiple aspects of the TME in response to these microenvironmental variations [146]. Hypoxia serves as a microenvironmental signal inducing the polarization of TAMs into a phenotype supporting tumor progression and can contribute to the development of therapy resistance [59]. A wide range of factors stimulate migration, including VEGF, chemokine ligand 2 (CCL2), chemokine ligand 5 (CCL5), colony-stimulating factor 1 (CSF-1), endothelial monocyte-activating polypeptide II (EMAP-II), endothelin-2, semaphorin 3A (SEMA3A), oncostatin M, and eotaxin, are produced in tumor hypoxic microenvironment leading to massive infiltration and entrapment of macrophages in these oxygen-deficient areas [59, 99]. TAMs disrupt antigen presentation, by secreting cytokines and inflammatory mediators like IL-10, TGF-*β*, prostaglandin E2 (PGE2) and matrix metalloproteinase 7 (MMP-7), thereby depriving T cells of the ability to distinguish or even kill tumor cells and moreover create an immunosuppressive microenvironment. Among them, TGF-*β* and IL-10 are dominant drivers of the immunosuppressive microenvironment in tumors [97]. Hypoxic TAMs promote tumor growth by enhancing the expression of growth factors, such as VEGF, PDGF, and fibroblast growth factor-2 (FGF-2) supporting development of neovasculature within hypoxic areas of the tumor [59]. Hypoxic TAMs also secrete distinct matrix metalloproteinases (MMPs), such as MMP-2, -9 that in different cancers negatively modulate inflammatory reactions [103]. In addition, M2 TAMs have the ability to hinder the effectiveness of immunotherapy by suppressing T cell activity and upregulating the expression of programmed death-ligand 1 (PD-L1) in the TME. Specifically, M2 TAMs impede the function of programmed cell death protein 1 (PD-1)/PD-L1 inhibitors by releasing anti-inflammatory cytokines and exosomes, thereby enhancing the presence of immune checkpoint ligands on the cell surface [112].

Understanding the role of hypoxia in TAM polarization is crucial for the development of effective immunotherapeutic strategies. Targeting hypoxia-associated signaling pathways in TAMs may help overcome the immunosuppressive effects and improve anti-tumor immune responses. Furthermore, combining therapies targeting TAMs with immune checkpoint inhibitors or other immunomodulatory agents may hold promise for improving treatment outcomes in cancer [148].

### Endothelial cells (ECs)

Tumor growth relies on the establishment of its own blood supply, known as vasculature. Tumor blood vessels exhibit abnormalities compared to normal vasculature. These abnormalities include the presence of abnormal ECs, pericytes, and basement membranes. The ECs in tumor blood vessels do not tightly adhere to one another, leading to loose intercellular junctions [131]. The chaotic and leaky blood vessels within the TME play a pivotal role in the formation of underoxygenated areas. These blood vessels exhibit abnormal architectural patterns, with irregular diameters and inconsistent branching, leading to compromised blood flow dynamics [89]. Consequently, the disrupted blood supply and impaired oxygen delivery create a hostile environment characterized by insufficient oxygen levels, ultimately promoting the development of hypoxic regions within the tumor.

ECs are critical in the functioning of various organs throughout the body as they are in direct contact with blood. Consequently, ECs are highly responsive to hypoxia and HIFs, which allow them to regulate essential processes such as cell survival, growth, cell invasion, and energy metabolism. When exposed to hypoxia, they undergo various metabolic adaptations (including increased glycolysis, biosynthesis of amino acids, carbon metabolism, pentose phosphate pathway, fructose/mannose, cysteine/methionine metabolism, and upregulation of genes involved in pyruvate metabolism and glucose transport). In addition to the direct effects of hypoxia on ECs, there are numerous indirect consequences, including the accumulation of lactate in the tumor environment, and some suggest they may play role in angiogenesis, maintaining development of tumor vascular system [119].

Under hypoxic conditions, the most important factor driving hypoxia, HIF-1, becomes stabilized and activates a variety of downstream genes involved in angiogenesis. Each step of vascularization is supported by the HIF-1. Vascular endothelial growth factor isoforms (VEGF-A, VEGF-B, VEGF-C, VEGF-D and EG-WVEGF) are basic factors that facilitate angiogenesis [13]. Hypoxia-induced angiogenesis, driven by HIF-1, is largely reliant on VEGF, primarily due to the fact that HIF-1 acts as a key stimulator of vascular endothelial growth factor production. In hypoxia VEGF binds with its receptors, initiating EC proliferation, migration, and tube formation. It may also enhance expression of other pro-angiogenic factors such as placental growth factor (PlGF) and FGF. Sufficient oxygenation of tumor tissue would stop the cascade [159]0.4.4. Tumor-infiltrating lymphocytes (TILs).

TILs are immune cells that infiltrate the tumor and play a critical role in anti-tumor immune responses. However, the hypoxic microenvironment poses significant challenges for the function and survival of TILs [28]. HIF-1*α* and its negative regulators have been recognized to play an important role in T cell activation and expansion [54]. Under hypoxic conditions, the expansion of therapeutic cells can enhance their tumor-killing capacity [35]. Despite the potential benefits of HIF-1*α* activation in certain contexts, hypoxia overall creates an immunosuppressive environment that can hinder immune responses, including T cell function and efficacy. In hypoxia, along with persistent antigen stimulation, T cell proliferation and effector functions are suppressed, resulting in diminished effectiveness of therapies [122]. Decreased oxygen levels in the TME can inhibit the production of crucial cytokines, such as IFN-*γ*, which is crucial for anti-tumor immune responses [87]. Furthermore, one of the key effects of hypoxia on T cells is the induction of a regulatory T cell (Treg) phenotypes. Tregs are immunosuppressive T cells that play a critical role in maintaining immune tolerance and preventing excessive immune responses. In hypoxic conditions, T cells upregulate the expression of HIF-1*α*, which drives the expression of Foxp3 and favors Treg stability [101].

The increased abundance of Tregs within the TME contributes to immunosuppression, limiting the activity of effector T cells and facilitating tumor immune evasion [39, 124]. Moreover, hypoxia can also promote the differentiation of T cells toward an exhausted or dysfunctional phenotype. Exhausted T cells are characterized by the progressive loss of effector functions, including decreased cytokine production and cytotoxicity. This exhaustion phenotype is often associated with sustained exposure to antigen and chronic immune stimulation. In the hypoxic TME, the expression of inhibitory receptors, such as PD-1, Tim-3 (T cell immunoglobulin and mucin domain-containing protein 3), and Lag-3 (lymphocyte activation gene 3), is upregulated on T cells. Consequently, exhausted T cells become less responsive to anti-tumor signals, impairing their ability to control tumor growth [8, 38].

In hypoxia, increased PD-L1 expression activates the Akt/mTOR pathway. Activation of immune checkpoints, including PD-L1 signaling through its PD-1 suppresses the Akt-mTOR pathway and reduces T cell glycolysis. Consequently, the TME impacts the metabolic abilities of infiltrating immune cells, leading to impaired anti-tumor effector function and facilitating tumor progression [75]. Conversely, hypoxia can also enhance the differentiation of a subset of T cells with pro-inflammatory and cytotoxic properties, known as Th17 (T helper 17) cells. Th17 cells produce interleukin-17 (IL-17) and other pro-inflammatory cytokines, promoting inflammation and recruiting immune cells to the TME. The balance between Tregs and Th17 differentiation is a critical determinant of tumor immune responses [32, 135].

### Cancer stem cells (CSCs)

CSCs share similar properties with normal stem cells. However, CSCs are a subpopulation of tumor cells possessing self-renewal properties and differentiation capabilities. They contribute to tumor initiation, progression, and metastasis, and are often more resistant to currently available anticancer therapies [67].

The hypoxic TME has been shown to have a profound impact on the maintenance and behavior of CSCs. Activation of HIF-1*α* and HIF-2*α* in hypoxic conditions can enhance the expression of genes associated with stemness, self-renewal, and pluripotency. These genes include Oct4, Sox2, Nanog, c-Myc, maintaining crucial properties of CSCs, through, among others, the activation of the Notch signaling pathway [43, 47, 145, 157]. Hypoxic conditions within the TME also promote the generation of high levels of ROS leading to activation of stress signaling pathways in CSCs through the TGF-*β* and tumor necrosis factor alpha (TNF*α*) signaling pathways. This helps to maintain the undifferentiated state of CSCs. Hypoxia within the TME creates selective conditions promoting the survival and expansion of CSCs. The hypoxic environment supports enhanced stemness of CSCs [5].

Hypoxia plays a critical role in maintaining CSCs in a quiescent state by influencing various cellular processes such as metabolic adaptation, apoptotic pathways, cell cycle regulation, and self-renewal. Quiescence, characterized as a protective response to adverse conditions, allows cells to preserve their proliferative potential and repair DNA damages [26]. The presence of non-dividing quiescent CSCs, which can survive conventional therapies that target rapidly dividing cells, is responsible for the failure of cancer treatments and the recurrence of tumors [38]. In hypoxic conditions causing acidification of the cells’ extracellular environment, acidic stress has been found to promote CSC-like phenotypes [25]. When CSCs derived from glioblastoma or CSC-depleted cultures of glioblastoma cells were exposed to low pH conditions, there was an upregulation of CSC markers such as OLIG2, OCT4, and NANOG. This suggests that acidic stress can contribute to the maintenance and expansion of CSC populations, potentially influencing tumor progression and treatment outcomes [61].

Notably, hypoxic conditions increase the cancer stem-like cell (CSC) fraction within the tumor microenvironment. This subpopulation is believed to be particularly aggressive and resistant to conventional therapies, making them a critical target for next-generation treatments. Some researchers suggest that CSCs are responsible for uncontrolled tumor growth and recurrence [5]. Given their resistance to therapies such as chemotherapy and radiotherapy, targeting these regions is crucial for inhibiting tumor growth and preventing recurrence.

### Natural killers (NK)

NK cells are a primary component of the innate immune system and play critical role in the recognition and elimination of tumor cells. NK can directly detect and kill target cells, including tumor cells with downregulated expression of major histocompatibility complex (MHC-I), without prior sensitization [154]. In response, they rapidly produce cytokines and secrete various immune molecules, including IFN-*γ*, TNF*α*, GM-CSF, and chemokines. These immune factors play a crucial role in activating other immune cells, such as T and B cells, thereby boosting the adaptive immune response [133].

However, the hypoxic TME can significantly influence the function and activity of NK cells, leading to altered anti-tumor responses.

Teng et al. [138] proved that hypoxia can dampen the cytotoxic activity of NK cells. It reduces their ability to secrete cytotoxic molecules, such as perforin and granzyme B, which are crucial for inducing target cell death. They observed a decrease in the expression of the IFN-*γ* cytokine, in hypoxia comparing to the normoxic condition. Additionally, the hypoxic environment led to a reduction in the expression of CD107a, a marker associated with the degranulation and activity of NK cells. They also confirmed that hypoxia has the ability to reduce the expression of activating receptors, such as NKp46, NKp30, and NKG2D, on the surface of NK cells. It is suggested that hypoxia can modulate the cytokine production profile of NK cells and can lead to a shift in cytokine production toward immunosuppressive factors, such as IL-10 and TGF-*β* [46]. CD73 expression has been shown to be induced by HIF-1*α* in various cell types, suggesting that a similar mechanism may occur in NK cells as well. Increased expression of CD73 on NK cell surfaces leads to the activation of STAT3 and secretion of TGF-*β*/IL-10, resulting in a correlation with reduced proliferation of CD4 + T cells and decreased production of IFN-*γ* [102]. NK cells constitutively express certain inhibitory receptors, such as killer-cell immunoglobulin-like receptor (KIR) and NKG2A, which play a role in regulating NK cell tolerance toward healthy tissues. In contrast, inhibitory receptors like PD-1 are typically expressed at low levels in NK cells from healthy donors but increase in pathological conditions [130]. However hypoxia can upregulate the expression of inhibitory receptors KIRs and PD-1, on NK cells. The increased expression of these inhibitory receptors can impair NK cytotoxicity [106]. Hypoxia can also affect the migration and infiltration of NK cells into the TME. It can disrupt the expression of chemokine receptors and adhesion molecules on NK cells, which are crucial for their recruitment and infiltration into tumor tissues. This impaired migration can limit NK cell access to hypoxic tumor regions, reducing their anti-tumor efficacy [2, 95].

## Hypoxia detection

**Fig. 3:**
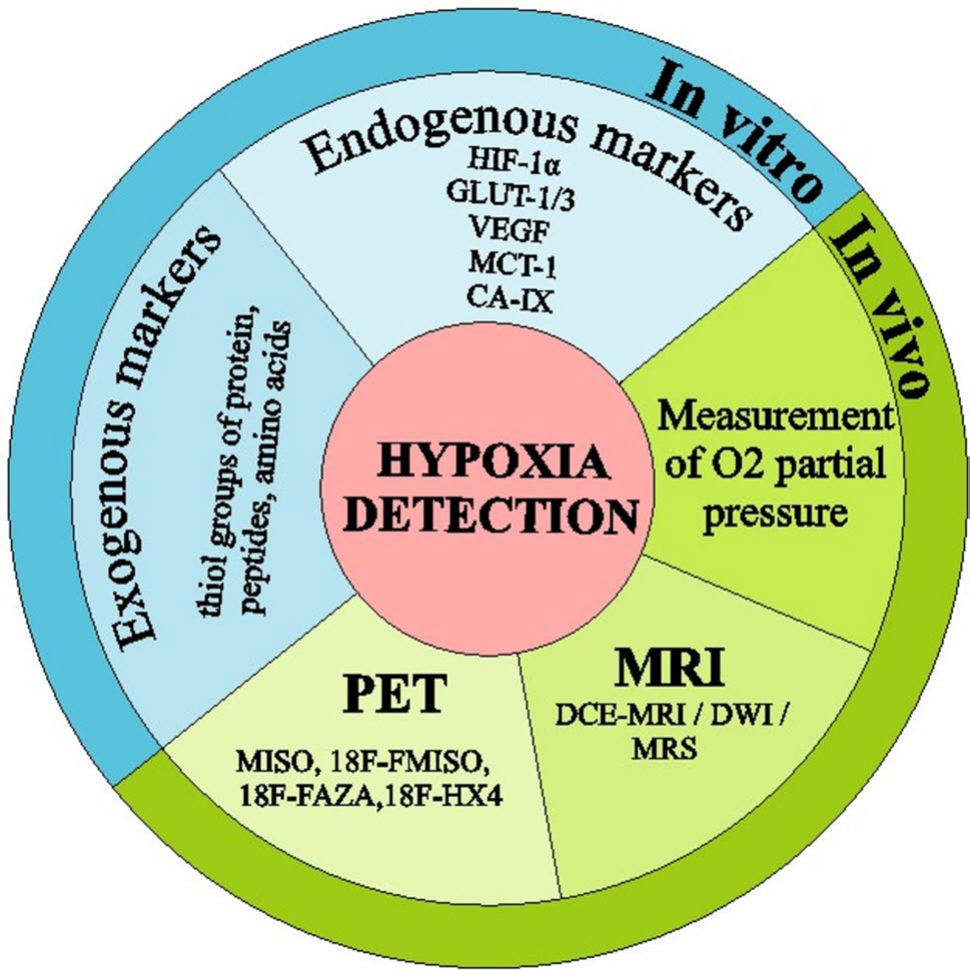
Detection of hypoxia in tumors. Hypoxia in tumors can be detected in vitro by determination of exo- and endogenous markers and in vivo by measurement of O_2_ pressure, and MRI and PET imaging

Accurate and reliable detection of hypoxia within the TME is crucial for understanding its role in tumor progression and therapeutic responses. Hypoxia, a hallmark feature of solid tumors, is associated with aggressive tumor phenotypes, increased metastatic potential, and resistance to conventional therapies. Detecting hypoxic areas within tumors is essential for identifying patients who may benefit from hypoxia-targeted therapies and for optimizing treatment strategies. In recent years, significant advancements have been made in developing various techniques to assess and visualize hypoxia in tumors noninvasively and with high spatial resolution. In this section, we will discuss some of the most relevant and cutting-edge methods for the detection and assessment of hypoxia in the TME (Fig. 3).

### Hypoxia detection in vitro

The simplest way to investigate effects of reduced oxygen on cell culture is to decrease oxygen levels to around 1% in an environment-controlled incubator. This strategy is useful while identifying the effects of hypoxia on 2D cell gene and protein expression, proliferation and viability. Although very informative, the monolayer model lacks a variety of other physiological gradients present in tumor hypoxic conditions observed in 3D structures, such as significant decrease in nutrients availability, buildup of lactate, and acidic pH, that impact microenvironment. Spheroids naturally establish an oxygen gradient, thereby providing a more precise visualization of a real tumor. Popescu et al. highlighted the difference between 2 and 3D cell models while researching cancer treatment. The study investigated the efficacy of nanoparticles (NPs) for doxorubicin (DOX) delivery and radiosensitization in 2D and 3D cell models of normal and tumor cells. In 2D cultures, NPs demonstrated varying cytotoxic effects on different cell types. Notably, in 3D models, the radiomodulating effect was influenced by spheroid morphology, with cancer cell 3D models showing radiosensitization and normal cell 3D models displaying a protective effect against ionizing radiation. This highlights the significance of utilizing three-dimensional models to analyze the intricate relationship between NP behavior and the response to radiation, offering insights for potential advancements in cancer treatment [110]. To identify the formation of the hypoxic regions, Close et al. fixed multicellular spheroids cultured under normoxic conditions (21% O_2_) and stained them using, among others, pimonidazole, HypoxiTRAK^™^ and monoclonal antibodies (mAb) detecting HIF-1*α*. Results showed that fluorescence of hypoxic regions within the center of assembled clusters of Head and Neck Squamous Cell Carcinoma (HNSCC) was observed [27].

#### Endogenous markers

HIF-1*α* immunolabeling have been frequently selected to identify hypoxia in cell cultures. However, the most challenging aspects appear to be the short half-life of the protein when reoxygenated and their localization in cell nucleus while in hypoxia, resulting in the necessity for nuclear permeabilization while staining.

The demand for readily accessible targets for immunolabeling, such as HIF-transcriptional targets (including glucose transporters 1/3 (GLUT-1/3), VEGF, monocarboxylate transporter 4 (MCT-1), and carbonic anhydrase IX (CA-IX)), highlights the necessity for further investigation [20, 52, 96].

Immunolabeling of HIF-1 and its downstream targets can be employed for the detection of hypoxia in cells exposed to low oxygen levels. The main drawbacks of these measurements are the inability to provide precise O_2_ concentration in tumors, and the need for tissue or cell fixation. It is also crucial to keep in mind that overexpression of HIFs may be induced by a variety of other factors than hypoxia [48].

#### Exogenous markers

Another approach to detect and visualize hypoxia within tumor tissues is immunolabeling of exogenous markers. This technique targets specific markers that accumulate in hypoxic regions and allows for the spatial identification and quantification of hypoxic areas within the TME. In hypoxia, a probe such as nitroimidazole can bind to various macromolecules including proteins, peptides, and amino acids, forming adducts with their thiol groups. The reduction of the imidazole ring allows for selective accumulation in hypoxic cells. To visualize hypoxic cells, a tag is introduced via an antibody that specifically recognizes the metabolic product of nitroimidazole. This allows for the detection and identification of hypoxic cells through specific labeling and imaging techniques [143]. Pimonidazole and EF-5 and specific antibodies (Hydroxyprobe^™^, ELK3-51) are widely used pairs to detect hypoxic areas, both in vitro and in vivo [3, 79, 118, 129]. Exogenous hypoxia markers, such as nitroimidazole derivatives, are valuable tools for detection of hypoxia. Although their use involves pre-exposure of the samples to the marker for an extended period, typically several hours followed by fixation and immunolabeling, these probes are not suitable for real-time measurements as they require sample preparation and staining procedures. Despite these limitations, they remain important tools in research and clinical settings for identifying and studying hypoxic regions within tissues or cell cultures.

### Hypoxia detection in vivo

The assessment of hypoxia in the TME is essential for understanding its impact on cancer progression and therapy response. In vivo experiments offer several key advantages over in vitro studies when detecting hypoxia within tumors. Unlike in vitro cell cultures, in vivo models better replicate the complex TME, taking into account factors such as cell–cell interactions, immune responses, and blood supply. This physiological context allows for a more accurate representation of how hypoxia develops and evolves in solid tumors, providing critical insights into its spatiotemporal distribution.

Imaging techniques have become an increasingly valuable form of noninvasive assessment of tumor biology. These techniques provide a comprehensive evaluation of cancer phenotype by offering information about the tumor’s size, shape, and location, as well as its functional and metabolic characteristics of TME [77]. This multidimensional approach enables a better understanding of tumor behavior, response to treatment, and overall prognosis. Among many imaging techniques, most commonly employed are measurement of O_2_ partial pressure, magnetic resonance imaging (MRI) and positron emission tomography (PET) [68].

#### Measurement of O2 partial pressure

Widely recognized as the “golden standard” to measure the distribution of the partial pressure of oxygen (pO_2_) is to use polarographic electrodes. They provide direct and localized measurements, allowing for precise assessment of oxygen levels at specific sites. Additionally, they offer real-time monitoring, enabling dynamic analysis of oxygen fluctuations. However, there are some limitations to consider. Invasive insertion of electrodes may disrupt tissue integrity, and the measurement itself can cause tissue damage. Moreover, the assessment of pO_2_ with a polarographic electrode can only be applied to superficially localized tumors [29].

#### Magnetic resonance imaging (MRI)

MRI is a versatile imaging modality that can provide detailed pathophysiological information about tumors. It allows for the assessment of various aspects of tumor biology and reflect changes in structure, metabolism, hypoxia, and acidic microenvironment [150]. MRI can provide anatomical, high–Resolution images showing the size, form, and location of tumors, enabling precise tumor staging and evaluation of tumor response to therapy. Herein, a selection of widely recognized MRI modalities, encompassing dynamic contrast-enhanced MRI (DCE-MRI), diffusion-weighted imaging (DWI), blood oxygen-level dependent (BOLD) MRI, and magnetic resonance spectroscopy (MRS), is presented. Collectively, these methodologies afford extensive insights into diverse tumor attributes, encompassing vascularity, perfusion, hypoxia, and metabolic modifications. Advanced MRI procedures such as DCE-MRI and DWI can also provide information on the cellularity, vascularity, and perfusion of tumors. These methods may be used to assess the angiogenesis, metabolism, and hypoxia in tumors [73]. DWI is a functional MRI technique that measures the random mobility of water molecules within tissues at the cellular level. By acquiring multiple images with different diffusion weightings, “apparent” diffusion coefficient (ADC) maps can be generated allowing for differentiation of hypoxic areas [140]. DCE-MRI involves administration of a contrast agent, typically gadolinium chelates. By tracking the uptake and washout of the contrast within the tumor, DCE-MRI provides information about tumor vascularity, permeability and blood flow, stromal cellularity and fibrosis, proliferation, and tissue oxygenation. Hypoxic regions may exhibit altered contrast enhancement patterns [80]. BOLD MRI relies on the paramagnetic properties of deoxyhemoglobin to detect changes in oxygenation levels. Tumor hypoxia leads to an increase in deoxyhemoglobin content, resulting in alterations in the magnetic properties of tissues [19]. MRS allows us to detect metabolic changes and concentrations of metabolites within tissues. Hypoxic regions in tumors often exhibit metabolic alterations, including increased levels of lactate due to anaerobic glycolysis [51]. However, MR imaging also allows us to measure the distribution of pyruvate, alanine, and bicarbonate in tumor tissue [70].

#### Positron emission tomography (PET)

In the early 1980s, a radionucleotide marker of hypoxia in tumors, misonidazole (MISO), was introduced [15]. MISO is a compound that selectively accumulates in hypoxic regions of tumors due to its chemical properties. A significant number of PET tracers have been developed specifically for the identification and imaging of hypoxia in living tissues and solid tumors. These tracers are designed to selectively accumulate in hypoxic regions and can provide valuable information about the oxygenation status of tumors. Some of commonly used PET tracers for hypoxia imaging include: ^18^F-fluoromisonidazole (^18^F-FMISO), ^18^F-fluoroazomycin arabinoside (^18^F-FAZA), and ^18^F-flortanidazole (^18^F-HX4), the three most extensively studied and commonly used tracers for hypoxia imaging [41]. None of the currently available tracers are perfect for imaging hypoxia in all tumor types. Studies have demonstrated the feasibility for imaging hypoxia in various cancers. One of the challenges with PET imaging for hypoxia is that the tracers used are not exclusively influenced by hypoxic conditions. While these tracers preferentially accumulate in hypoxic areas, other factors can also contribute to their uptake and retention within tissues. For example, some tracers may show non-specific binding or accumulation in regions with high metabolic activity or inflammation, which can lead to false-positive results or confound the interpretation of the imaging data [68].

## Potential use of hypoxia in anti-*cancer* therapies

Regions of low oxygen in microenvironments within solid tumors have been recognized as a potential target for therapeutic interventions. The unique characteristics of hypoxia offer tremendous opportunities for developing strategies that specifically target and exploit these regions. Here are some potential therapeutic approaches that leverage hypoxia in cancer treatment.

### Hypoxia-activated prodrugs (HAPs)

HAPs are designed to selectively release cytotoxic agents specifically within hypoxic regions of tumors. These prodrugs remain inactive under normoxic conditions and have little toxicity to normal tissues. They undergo bioreduction in the presence of low oxygen levels, releasing cytotoxic compounds that can induce cell death in hypoxic tumor cells. This group of compounds includes quinones, nitroaromatics, aliphatic N-oxides, and hetero-aromatic N-oxides. Among them, the most prominent and representative ones are tirapazamine, AQ4N (banoxantrone), PR-104, EO9 (apaziquone), TH-302 (evofosfamide), and SN30000. HAPs offer the potential for targeted therapy by exploiting the hypoxic environment within tumors while sparing surrounding healthy cells [22, 82]. In a study, Alexander van der Wiel et al. [142], evaluated CP-506, a DNA alkylating hypoxia-activated prodrug, selectively targeting hypoxic tumor cells. It resists aerobic metabolism and demonstrates broad anti-tumor activity, inhibiting growth in various hypoxic xenografts. A novel NP, iRGD@ZnPc + TPZ, introduced by Zhang et al., for enhanced glioma treatment by encapsulating the hypoxia-activated prodrug tirapazamine (TPZ) and the photosensitizer zinc phthalocyanine (ZnPc). Overcoming the challenges posed by insufficient hypoxia and the blood–brain barrier (BBB). Through iRGD-mediated receptor targeting, the nanoparticle breaks the BBB, achieves deep penetration, and significant retention in gliomas. The photosensitizer-enhanced activation of TPZ within the gliomas presents a promising strategy for amplified chemotherapy, addressing a critical need in clinical glioma treatment [155].

### Oxygen-enhanced therapies

Oxygen-enhanced therapies aim to elevate the blood pO_2_ concentration and improve oxygen delivery to hypoxic tumor regions independent of hemoglobin. The supply of oxygen to the tissue can be achieved through physical dissolution of the oxygen in the blood plasma that reaches hypoxic regions [49]. This approach can increase the effectiveness of radiation therapy (RT) and chemotherapy [12, 94]. This can be achieved through various approaches, such as hyperbaric oxygen (HBO) therapy, where patients are administered with pure oxygen under increased atmospheric pressure. HBO therapy increases oxygen availability to tumor tissues and can enhance the efficacy of radiation therapy and certain chemotherapeutic agents. HBO therapy can activate immune response against cancer cells [86]. However, it might also cause serious side effects when oxygen accumulates in healthy tissues, such as barotrauma, hyperoxic seizures and ROS-mediated cytotoxicity [9]. Another approach involves the use of oxygen-generating or oxygen-carrying nanocarriers, such as catalase (CAT) and perfluorocarbons (PFCs), to produce or deliver oxygen directly into hypoxic regions, improving tumor oxygenation and sensitizing tumor cells to treatment [65, 81, 149].

### Hypoxia-targeted photodynamic therapy (PDT)

Hypoxia-targeted PDT is a treatment that uses the combination of light at an appropriate wavelength, a specific anti-hypoxia photosensitizer (PS), and oxygen to selectively destroy tumor cells. Photosensitizing agents are administered to the patient, and upon activation, will generate cytotoxic ROS that cause localized cell death and tissue damage [23, 36, 66]. Lin et al. presented a novel photosensitizer, Ion-BDP, to enhance efficacy of PDT against hypoxic tumor cells. Regulated by nitroreductase (NTR), it generates ROS for aerobic tumor cells and converts to a potent photothermal agent (BDP) under hypoxic conditions both in vitro and in vivo [85]. Wen, with colleges, showcased encapsulation of the PS IR780 and oxygen regulator 3-bromopyruvate (3BP) in poly-lactic-co-glycolic acid) (PLGA) nanocarriers, addressing the challenges of hypoxia and poor PS accumulation in tumors during PDT. This nanoplatform, demonstrated in animal models, achieves deep tumor penetration, mitochondria targeting, and efficient ROS generation through simultaneous reduction of oxygen consumption and inhibition of glycolytic capacity, presenting a promising strategy for enhanced cancer treatment with imaging guidance [151].

### Immunotherapy targeting hypoxia

Immunotherapy has revolutionized cancer treatment by harnessing the body’s immune system to recognize and eliminate cancer cells. However, the hypoxic TME poses challenges to immune responses and can contribute to immune evasion. Targeting hypoxia in combination with immunotherapy has emerged as an innovative approach to enhance the effectiveness of immune-based treatments. Modulating HIF signaling that regulates the expression of various genes involved in angiogenesis, metabolism, and immune responses, which could allow to overcome immunosuppression of hypoxic TME. A combination of immune checkpoint inhibitors, such as anti-PD-1/PD-L1 antibodies, with hypoxia-targeted therapies could disrupt evasion immune response by upregulation of immune checkpoint molecules, such as PD-L1, that dampens function of CD8^+^ T cells [44]. Most promising are combinations of immunotherapy with hypoxia-targeted therapies; while, they hold potential for synergistic effects.

Jayaprakash et al. [72] showcased that the hypoxia-activated prodrug TH-302 effectively diminishes or eradicates hypoxia within prostate tumors. In a notable finding, the combination therapy involving this hypoxia-prodrug and checkpoint blockade of CTLA-4 and PD-1 demonstrated a cooperative effect, resulting in the cure of over 80% of tumors in the transgenic adenocarcinoma of the mouse prostate-derived (TRAMP-derived) TRAMP-C2 model. The company ImmunoGenesis announced the initiation of a Phase 2 clinical trial in 2021, exploring the use of evofosfamide (hypoxia-activated prodrug) as a hypoxia-reversal agent in conjunction with both CTLA-4 and PD-1 blockade. This trial is focusing on patients diagnosed with castration-resistant prostate cancer (CRPC), pancreatic ductal adenocarcinoma (PDAC), and HPV-negative HNSCC [58].

### Hypoxia-targeted drug delivery with nanocarriers

Nanotechnology has emerged as a promising approach to improve the delivery of anticancer therapies, especially in the context of targeting hypoxic regions within tumors. Hypoxia is a major challenge in cancer treatment, as it promotes therapy resistance and contributes to tumor aggressiveness. Traditional anticancer drugs often have limited access to hypoxic regions due to compromised tumor vasculature and reduced drug perfusion. Nanovehicles and NPs offer unique advantages in overcoming these barriers and enhancing therapeutic efficacy [24].

Nanovehicles, such as liposomes, polymeric NPs, and micelles, can encapsulate anticancer drugs and shield them from premature degradation and clearance. These nanocarriers are engineered to passively accumulate within the TME through the enhanced permeability and retention (EPR) effect. The EPR effect exploits the leaky vasculature and impaired lymphatic drainage characteristic of solid tumors, enabling nanovehicles to preferentially accumulate in hypoxic regions [153]. As a result, these nanovehicles can deliver higher drug concentrations to tumor areas, where conventional therapies often fail to penetrate effectively [16].

Furthermore, hypoxia-targeted NPs can be designed to respond to specific triggers found within hypoxic regions. For instance, the hypoxic TME exhibits distinct physiological characteristics, such as low pH, high levels of reducing agents, and elevated enzymatic activity. NPs engineered with hypoxia-sensitive moieties can respond to these cues and trigger drug release selectively within hypoxic areas.

Son et al. [132] developed a hypoxia-responsive NP system using a polymer conjugate of carboxymethyl dextran (CMD) and black hole quencher 3 (BHQ3). The created delivery system, forming CMD-BHQ3 NPs, exhibiting sustained release of DOX under normal conditions with significantly increased release under hypoxic conditions. Feng et al. synthesized a novel diblock polymer of polyethylene glycol and poly[glutamic acid (3-(2-nitro-imidazolyl)-propyl)], forming hypoxia-responsive polymeric micelles for controlled DOX release. In vitro experiments showcased enhanced apoptosis of tumor cells under hypoxic conditions by DOX-loaded micelles [40]. To address hypoxia in breast cancer microenvironments, Mamnoon synthesized estradiol-conjugated hypoxia-responsive polymeric nanoparticles (E2-Dox-HRPS) for targeted drug delivery. These polymersomes released over 90% of encapsulated DOX within hypoxia, displaying higher internalization and cytotoxicity in estrogen receptor-positive breast cancer cells compared to non-targeted polymersomes. The novel E2-Dox-HRPS presents a promising strategy for targeted drug delivery in estrogen receptor-positive breast cancer therapy, particularly in hypoxic conditions [90]. Abdullah designed covalent conjugation of DOX to a pH-sensitive nanocarrier (PCPY-cDOX) which significantly reduced cardiotoxicity in primary cardiomyocytes compared to noncovalently encapsulated DOX NPs (PCPY-eDOX). PCPY-cDOX exhibited prolonged control over drug release at an acidic pH level, preventing cardiotoxicity by impeding the nuclear entry of the drug. This approach maintained chemotherapeutic efficacy while minimizing cardiotoxic effects, highlighting the importance of nanocarrier selection in optimizing the balance between low overall toxicity and therapeutic potency [1].

This controlled drug release ensures that the anticancer drug is delivered precisely to the regions where it is most needed, minimizing off-target effects and reducing systemic toxicity.

Moreover, NPs hold promise as carriers for combination therapies that simultaneously target hypoxia and other therapeutic resistance mechanisms. By co-loading different anticancer agents or therapeutic modalities, NPs can achieve synergistic effects and overcome the challenges posed by the heterogeneous and dynamic nature of the hypoxic TME.

Lee et al. developed hypoxia-responsive NPs releasing chlorin e6 (Ce6) and paclitaxel (PTX) selectively under hypoxic conditions. Functionalized with 4.4′-azodianiline (Azo) and RGD-conjugated poly(ethylene glycol), these NPs exhibited uniform Ce6 distribution within HeLa cells and spheroids, enhancing anti-tumor activity in hypoxic environments. The strategy demonstrated significant anti-tumor effects in a HeLa cell xenograft mouse model [78]. Li et al. [83] demonstrated a potent synergistic effect of Icaritin with the epigenetic drug JQ1 in suppressing breast cancer, mitigating drug resistance. Enhancing tumor-targeted efficacy, a hypoxia-cleavable, RGD peptide-modified PLGA-NP (ARNP) was developed for the targeted delivery of JQ1 and icaritin. In vitro and in vivo assessments revealed effective biodistribution, primary tumor, and bone metastasis suppression, along with mitigation of bone erosion. ARNP also significantly inhibited pulmonary metastasis secondary to bone metastasis (Table 1).

**Table 1:** Therapies using hypoxia as an effective target for cancer therapy

Type of therapy	Mechanisms	References
Hypoxia-activated prodrug	Bilirubin-chitosan conjugate (named as BR-Chitosan) with a HAP, nanovesicles that deplete oxygen in tumors to activate hypoxia-activated prodrugs and enhance photothermal therapy effectiveness	[22]
	CP-506, a DNA alkylating hypoxia-activated prodrug, selectively targeting hypoxic tumor cells, high cytotoxicity in low-oxygen conditions, resisting aerobic metabolism, reducing hypoxic fractions, inhibiting growth in various hypoxic xenograft models	[142]
	iRGD@ZnPc + TPZ targeting gliomas, enhancing hypoxia and activating hypoxia-activated prodrugs, overcoming blood–brain barrier and insufficient hypoxia	[155]
Oxygen-enhanced therapies	T1 mapping and oxygen-enhanced MRI (OE-MRI) for predicting radiotherapy outcomes in HPV-positive oropharyngeal cancer	[12]
	OE-MRI—well-tolerated technique for detecting hypoxia in head and neck squamous cell carcinoma (HNSCC) showing potential differences in oxygen responses between radiotherapy-responsive and resistant tumors	[94]
	HBO therapy enhances the anti-tumor efficacy of nanomedicine treatments, including photodynamic and photothermal therapies	[149]
	Perfluorocarbon-based polymer micelles delivering both a photosensitizer and oxygen, enhancing photodynamic therapy efficiency and reducing cell toxicity by improving singlet oxygen generation	[65]
Photodynamic therapy	Ion-BDP, a photosensitizer that switches to a photothermal agent in hypoxic conditions, enhancing photodynamic therapy by targeting both aerobic and hypoxic tumor cells	[85]
	PLGA-based nanoplatform encapsulating IR780 and 3-bromopyruvate enhancing photodynamic therapy by improving photosensitizer accumulation, reducing tumor hypoxia, and targeting mitochondria	[151]
Immunotherapy targeting hypoxia	Combination of hypoxia-activated prodrug TH-302 with immune checkpoint blockade overcomes resistance in prostate cancer by reducing hypoxia, increasing T cell infiltration, and decreasing myeloid-derived suppressor cells	[72]
	Combination of evofosfamide with ipilimumab in immunotherapy-resistant cancers showed manageable safety with common side effects including hematologic toxicities and elevated liver enzymes	[58]
Hypoxia-targeted drug delivery with nanocarriers	Hypoxia-responsive nanoparticles (CMD-BHQ3 NPs) loading and releasing DOX preferentially in hypoxia, showing increased cytotoxicity in hypoxic tumor cells and preferential tumor accumulation in vivo	[132]
	Hypoxia-responsive polymeric micelles (polyethylene glycol and poly[glutamic acid (3-(2-nitro-imidazolyl)-propyl)] diblock polymer) for controlled DOX release exhibited enhanced tumor cell killing under hypoxic conditions	[40]
	Hypoxia-responsive nanoparticles (E2-Dox-HRPs) for targeted drug delivery to estrogen-receptor-positive breast cancer released DOX preferentially in hypoxia, showing enhanced cytotoxicity and internalization compared to non-targeted nanoparticles	[90]
	Covalently linking doxorubicin to a pH-sensitive nanocarrier (PCPY–cDOX) significantly reduces cardiotoxicity compared to non-covalent encapsulation (PCPY–eDOX), while maintaining efficacy in breast cancer cell lines	[1]
	Hypoxia-responsive NPs releasing chlorin e6 (Ce6) and paclitaxel (PTX) selectively in hypoxia, showing enhanced anti-tumor activity in HeLa cells and xenograft models	[78]
	Hypoxia-cleavable, RGD peptide-modified poly(D,L-lactide-co-glycolide) (PLGA) nanoparticle (ARNP) delivering icaritin and epigenetic drug JQ1, effectively targeting breast cancer cells, suppressed primary tumors, bone metastasis, and pulmonary metastasis in vivo	[83]

## Conclusions

The hypoxic microenvironment within tumors plays a critical role in tumor progression, therapeutic resistance, and immune evasion. Understanding the impact of hypoxia on various cell types, such as cancer cells, CAFs, TAMs, ECs, TILs, and CSCs, provides valuable insights into the complex interplay between hypoxia and cancer biology. The detection and assessment of hypoxia have progressed, with a range of techniques now available to evaluate oxygen levels within tumors. These techniques include immunolabeling of exogenous markers, oxygen-sensitive electrodes, microelectrodes, and PET and MRI imaging.

Tumors are one of the few tissues characterized by extensive areas of hypoxia. These regions arise due to rapid tumor growth outpacing the supply of functional blood vessels, leading to oxygen deprivation. Low oxygen levels, in turn, are associated with abnormal blood vessel structure and function. Targeting tumor hypoxia is likely to become a key strategy in anticancer therapy. This could be achieved either by reducing hypoxia with hypoxia-reducing agents or by directly targeting these oxygen-deprived regions. Nanocarriers designed to aim at hypoxic regions present a promising strategy for targeted drug delivery. In a novel approach, we are developing a polymeric nanocarrier, or polymersome, loaded with two distinct anticancer agents: an immunostimulant targeting the STING pathway and a chemotherapeutic. These agents will be selectively released in hypoxic tumor regions following systemic administration.

Exploiting hypoxia as a potential target for cancer therapy has gained significant interest. HAPs offer selective cytotoxicity within hypoxic tumor regions, while modulating HIF signaling and overcoming immunosuppressive mechanisms within the hypoxic microenvironment has shown promise in enhancing the efficacy of immunotherapy. Oxygenation strategies, such as HBO therapy and oxygen-carrying agents, can improve tumor oxygenation and sensitize tumors to various treatment modalities, including radiation therapy and PDT. Additionally, combining immunotherapy with hypoxia-targeted approaches or developing hypoxia-specific immunotherapies holds potential for synergistic effects and improved treatment outcomes.

It also seems reasonable to identify hypoxic areas in tumors of patients undergoing radiotherapy, chemotherapy, or immunotherapy. Extensive hypoxic regions can significantly reduce the effectiveness of these treatments. The development of new methods to detect hypoxia or discover diagnostic markers that indicate the presence of such regions is crucial for enabling personalized cancer therapies that could significantly enhance patient outcomes. More precise, individualized treatment plans could optimize therapeutic success and potentially transform the standard of cancer care.

The comprehensive understanding of hypoxia in cancer biology and its therapeutic potential opens avenues for personalized and targeted cancer treatment strategies. However, further research is needed to optimize hypoxia-targeted therapies, identify biomarkers of hypoxia response, and overcome challenges associated with hypoxia-induced treatment resistance.

## Data Availability

No datasets were generated or analyzed during the current study.
